# Exosomes From Adipose‐Derived Mesenchymal Stem Cells: A New Prospective Therapy of Diabetic Foot Ulcer

**DOI:** 10.1155/bmri/6652338

**Published:** 2026-06-11

**Authors:** Rong Zhang, Zhanshuai Song, Juan Zhang, Wenwen Zhang, Linlin Li, Feifei Mou, Yongtao Su, Kai Mou

**Affiliations:** ^1^ Institute of Tissue Regeneration and Wound Repair, Institute of Pharmacy, Shandong University of Traditional Chinese Medicine, Jinan, Shandong, China, sdutcm.edu.cn; ^2^ Zibo No. 1 Hospital, Zibo, Shandong, China; ^3^ College of Traditional Chinese Medicine, Shandong University of Traditional Chinese Medicine, Jinan, Shandong, China, sdutcm.edu.cn; ^4^ Department of Medical Genetics, Zibo Maternity and Child Health Care Hospital, Zibo, Shandong, China; ^5^ Rehabilitation Department, Affiliated Occupational Disease Hospital of Shandong First Medical University, Jinan, Shandong, China; ^6^ Wound Repair and Plastic surgery, Affiliated Hospital of Shandong University of Traditional Chinese Medicine, Jinan, Shandong, China, sdutcm.edu.cn

**Keywords:** diabetic foot ulcer, exosomes, mesenchymal stem cell-derived exosomes, new therapy

## Abstract

Diabetic foot ulcer (DFU) is one of the most severe complications of diabetes, characterized by high rates of morbidity, disability, and mortality. Current treatment modalities for DFU primarily include surgical debridement, negative‐pressure wound therapy, and anti‐infection measures. However, these approaches are limited by prolonged treatment duration, high costs, and suboptimal therapeutic outcomes. Adipose‐derived mesenchymal stem cells (ADMSCs) have garnered significant attention in wound repair and tissue regeneration due to their ability to secrete a variety of cytokines involved in the healing process. Exosomes, as key mediators of the paracrine effects underlying the therapeutic benefits of ADMSCs, are emerging as promising agents for wound repair. As a novel therapeutic strategy, exosomes have been increasingly investigated for the treatment of DFU. In this review, we summarize the applications and therapeutic potential of ADMSC‐exosomes in DFU, aiming to provide a valuable reference for its clinical management.

## 1. Introduction

In diabetic individuals, diabetic foot ulcer (DFU) arises from varying contributions of peripheral vascular disease and neuropathy, leading to deep tissue ischemia, necrosis, ulceration, and subsequent infection in the lower limbs [1]. DFU represents a serious complication of diabetes, imposing substantial burdens on both patients and healthcare systems, and is associated with high rates of morbidity and mortality [2]. Once antimicrobial resistance develops or bacterial biofilms are established, conventional treatments often become ineffective, and amputation may become unavoidable. Globally, the prevalence of diabetes among adults aged 20–79 years was estimated at 10.5% (approximately 536.6 million individuals) in 2021, and is projected to rise to 12.2% (around 783.2 million) in 2045 [3]. It has been reported that approximately 19%–34% of diabetic patients will develop DFU during their lifetime, with a considerable proportion progressing to amputation [4]. In addition, DFU imposes a significant economic burden and further complicates the management of diabetes [5]. Current treatment strategies for DFU primarily include surgical intervention combined with anti‐infective therapy, hyperbaric oxygen therapy, negative‐pressure wound therapy, and minimally invasive procedures [6]. Nevertheless, these approaches are often limited by prolonged treatment duration, suboptimal efficacy, and high costs [7], which not only adversely affect patients′ physical and psychological well‐being but also impose considerable financial strain on healthcare systems. Therefore, there is an urgent need to develop more effective therapeutic strategies for DFU.

Recently, mesenchymal stem cells (MSCs) have attracted considerable attention due to their strong capacity to promote tissue regeneration and repair. Among them, adipose‐derived mesenchymal stem cells (ADMSCs) are considered particularly promising for therapeutic applications, as they can be obtained with minimal invasiveness, are abundant in source, and yield higher cell numbers compared with bone marrow‐derived MSCs [8, 9]. However, several limitations hinder the clinical translation of ADMSC‐based therapies, including potential risks of immune rejection and tumorigenesis, relatively low engraftment efficiency, and ethical concerns. Accumulating evidence indicates that the beneficial effects of MSCs are largely mediated through paracrine mechanisms, involving the secretion of growth factors, cytokines, and extracellular vesicles (EVs) [10]. Among these, exosomes represent a crucial component of the paracrine signaling and play an important role in tissue repair and regeneration. Exosomes exhibit several advantages, including low immunogenicity, high stability, ease of storage, and potential for large‐scale production from ADMSCs [11]. Therefore, exosomes derived from adipose‐derived mesenchymal stem cells (ADMSC‐exosomes) have emerged as a promising cell‐free therapeutic strategy for enhancing tissue repair and regeneration in DFU [10].

In this review, we summarize recent advances in the use of ADMSC‐exosomes for the treatment of DFU and systematically analyze the underlying mechanisms by which they promote wound healing and tissue repair. These insights will deepen our understanding of their therapeutic potential, facilitate the development of novel treatment strategies, and ultimately contribute to improved management and prevention of DFU.

## 2. Exosomes

**Figure 1:**
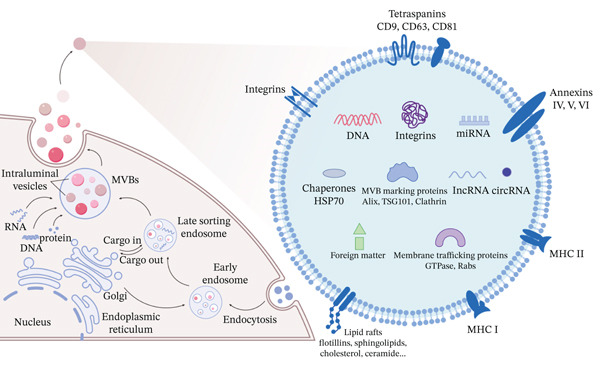
Overview of exosome generation and secretion.

As illustrated in Figure 1, exosomes are a subclass of EVs with diameters typically ranging from 30 to 200 nm. They originate from various somatic cells and are widely present in biological fluids, including blood and urine. Exosomes serve as important mediators of intercellular communication by delivering bioactive molecules such as mRNA, microRNA, protein, and lipids [10, 12]. With increasing recognition of their roles in both physiological and pathological processes, interest in exosome research has grown substantially. These vesicles carry diverse cargo derived from their parental cells, including proteins, nucleic acids, and lipids, which can modulate immune responses and inflammatory processes, thereby exerting either pathogenic or therapeutic effects [13]. Efficient isolation of exosomes is critical for investigating cell‐cell communication [14] and for their potential use as clinical biomarkers. Currently, commonly employed isolation techniques include ultracentrifugation, size‐exclusion chromatography, polymer‐based precipitation, ultrafiltration, commercial kits, and immunoaffinity capture assays. Among these, ultracentrifugation remains the most widely accepted “gold standard”, as it separates exosomes based on differences in size and density [15], and is valued for its relative simplicity and ability to produce high yields. Following isolation, exosomes are typically characterized using a combination of analytical methods, such as electron microscopy [16], nanoparticle tracking analysis [17], protein immunoblotting [18], flow cytometry [19], and nanoscale flow cytometry [20].

## 3. ADMSC‐Exosomes

Adipose tissue is now recognized not only as an energy reservoir but also as an active endocrine organ with diverse regulatory functions [21]. Exosomes have been detected in adipose tissue [22], as well as in adipocytes [23, 24] and ADMSCs [25]. In recent years, exosomes derived from ADMSCs have attracted increasing attention for their potential applications in skin wound healing and regenerative medicine [10]. Notably, the yield of ADMSCs obtained from adipose tissue is approximately 500‐fold higher than that from an equivalent volume of bone tissue [26, 27]. In terms of physical properties, ADMSC‐exosomes tend to be larger than conventional exosomes; however, they consistently express classical exosomal markers, including CD63 and HSP‐70, indicating that exosome size may vary depending on their cellular origin [28]. Moreover, EVs derived from adipocytes can be broadly divided into large extracellular vesicles (LEVs) and small extracellular vesicles (SEVs), each characterized by distinct proteomic profiles [29]. Specifically, LEVs are enriched in phosphatidylserine, whereas SEVs contain higher levels of cholesterol, suggesting that lipid composition is closely associated with the originating cell type [29, 30]. The high abundance of ADMSCs, together with the relative simplicity of their isolation, greatly enhances their practicality and applicability in clinical settings.

## 4. Pathogenesis of DFU

**Figure 2:**
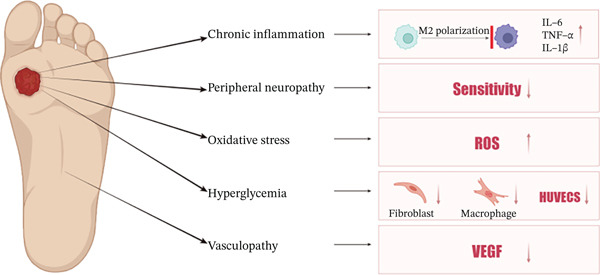
The pathogenesis of DFU.

Currently, the exact pathogenesis of DFU has not been fully clarified. As illustrated in Figure 2, its development is considered to be multifactorial, involving sustained hyperglycemia, oxidative stress (OS), chronic inflammation, vascular insufficiency, and peripheral neuropathy. OS is defined as an imbalance between pro‐oxidant and antioxidant systems, ultimately resulting in cellular dysfunction and tissue damage [31–33]. Excessive production of reactive oxygen species (ROS), together with impaired antioxidant defenses, leads to redox disequilibrium, which has been recognized as a key contributor to impaired wound healing in diabetic [34, 35]. Infection also plays an important role in the progression of DFU. Studies have identified a wide spectrum of pathogens, including gram‐positive bacteria such as *Staphylococcus aureus*, S*treptococcus agalactiae*, *Streptococcus dysgalactiae*, and *Enterococcus* spp., gram‐negative organisms such as *Pseudomonas aeruginosa*, *Escherichia coli*, *Klebsiella* spp., and *Proteus* spp., as well as anaerobes including *Bacteroides* spp. and *Peptostreptococcus* spp. [36, 37]. The hyperglycemic microenvironment in diabetic wounds facilitates microbial growth and toxin production, further impairing angiogenesis and delaying tissue repair. Therefore, strategies aimed at controlling inflammation and infection may promote wound healing in DFU [38]. DFU is fundamentally driven by the combined effects of peripheral neuropathy and vascular disease. It is estimated that more than half of the approximately 460 million individuals with diabetes worldwide are affected by neuropathy [39]. Disorders of carbohydrate, lipid, and protein metabolism render peripheral nerves more vulnerable to chronic compression injury [40], whereas persistent hyperglycemia contributes to demyelination and axonal degeneration of sensory nerve fibers [41]. Neuropathy, including sensory, motor, and autonomic components, represents a major pathogenic factor in DFU. Sensory neuropathy reduces the ability to perceive pain, temperature, and pressure, leading to unrecognized minor injuries that may progress to ulceration [42]. Among various risk factors, sensory neuropathy is considered the most prevalent contributor to DFU development [42]. Motor neuropathy further increases ulcer risk by causing progressive joint destruction, deformities, and chronic musculoskeletal damage [43]. Damage to motor nerve fibers can also lead to intrinsic muscle atrophy of the foot, impaired postural control, and reduced coordination, resulting in structural deformities such as prominent metatarsal heads and claw toes, which increase mechanical stress and susceptibility to skin breakdown [44]. In addition, autonomic neuropathy affecting sweat gland function reduces sweating, leading to dry and inelastic skin that is prone to fissures and secondary infection, thereby further compromising wound healing. Collectively, neuropathy remains one of the most important underlying mechanisms contributing to DFU [45].

## 5. ADMSC‐Exosomes in the Therapy of DFU

As key mediators in both physiological and pathological processes, ADMSC‐exosomes have attracted growing interest for their therapeutic potential in DFU. Recent studies on their application in DFU treatment are summarized in Table 1. Accumulating evidence suggests that ADMSC‐exosomes can effectively enhance angiogenesis and promote cell migration and proliferation, thereby accelerating wound closure and improving diabetes‐related pathological features. However, most current studies remain limited to in vitro experiments and rodent models, such as rats and mice. Clinical studies are still scarce, and robust validation is lacking, which limits the immediate translation of ADMSC‐exosome‐based therapies for DFU.

**Table 1:** ADMSC‐exosomes in the therapy of DFU.

Year	Author	Therapeutic effect	Target
2025	Liu et al. [46]	ADMSC‐exosomes enhanced human umbilical vein endothelial cell and fibroblast proliferation, migration, and tube formation.	In vitro
2025	Liu et al. [46]	ADMSC‐exosomes administration reduced ulcer size, increased angiogenesis (VEGF/CD31 expression), and decreased inflammatory markers (TNF‐*α*, IL‐6).	In a rat DFU model
2024	Yin et al. [47]	CircRps5 carried by ADMSC‐exosomes overexpression inhibited excessive autophagy, and when macrophages overexpressed miR‐124‐3p, the induction of the M2 phenotype was suppressed.	In high glucose‐induced macrophages
2025	Song et al. [48]	ADMSC‐exosomes enhance the proliferation and migration of fibroblasts under HG conditions, reduce excessive myofibroblast differentiation and collagen deposition, and promote scarless healing of diabetic wounds. Additionally, miR‐204‐5p in. ADMSC‐exosomes targets TGF‐*β*1 to inhibit p‐Smad2/3, Col I, and alpha‐smooth muscle actin (*α*‐SMA), thereby reducing fibrosis.	In rat skin fibroblasts in high‐glucose medium
2023	Song et al. [49]	ADMSC‐exosome treatment effectively reduces inflammation and promotes angiogenesis, collagen deposition, cell proliferation, and migration, thereby accelerating the wound healing process.	In vivo and in vitro
2025	Wang et al. [50]	ADMSC‐exosomes inhibited endothelial cell damage and promotes wound healing by targeting the miR‐199a‐5p/HIF1*α* axis.	In a mice DFU model
2025	Bai et al. [51]	The impact of ADMSC‐exosomes on mitochondrial function, autophagy flux, and inflammatory response were partially dependent on SIRT1.	In diabetic mice and high glucose‐treated macrophages
2025	Guo et al. [52]	ADMSC exosomes reduced inflammatory infiltration and promoted granulation formation and wound healing in wound tissues. ADMSC exosome injection increased the expression of CD34, Ki‐67, VEGF, and TGF*β*‐1, but decreased the expression of DLL‐4, TLR‐4, and IL‐6 in wound tissues of DFU rats. Injection of ADMSC exosomes reversed the negative effects of HG on the proliferation, migration, and angiogenesis of HUVECs, and enhanced cell apoptosis.	In rat models with DFU and human umbilical vein endothelial cells

## 6. Mechanism of Action of ADMSC‐Exosomes in Promoting Wound Healing in DFU

**Figure 3:**
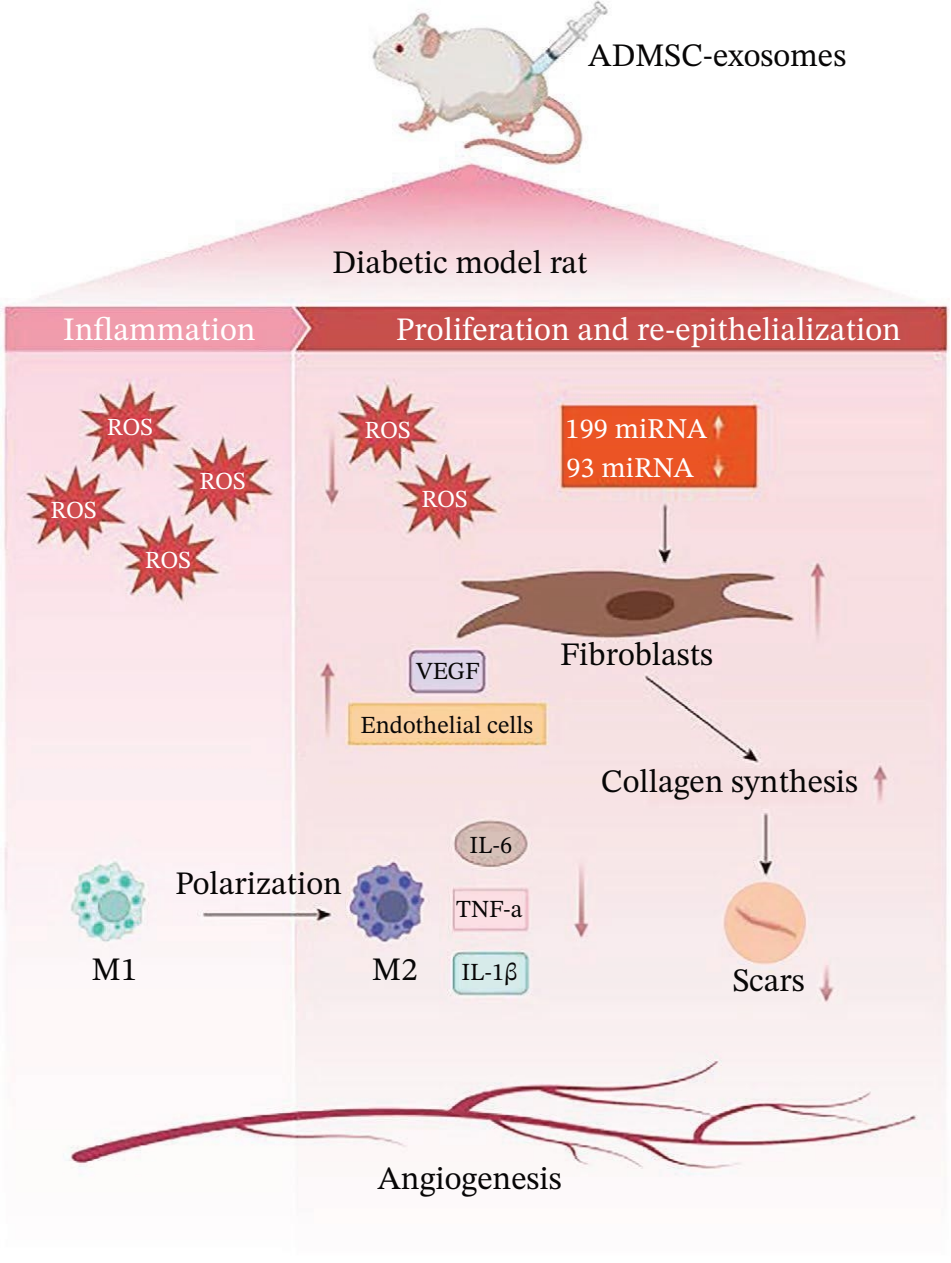
The mechanisms of action of the ADMSC‐exosomes in wound repair in diabetic mice.

As shown in Figure 3, ADMSC‐exosomes may contribute to DFU repair through a range of coordinated biological effects, including the suppression of OS, modulation of inflammatory and anti‐infective responses, enhancement of angiogenesis, stimulation of skin cell proliferation and re‐epithelialization, and regulation of collagen remodeling to limit excessive scar formation. A similar therapeutic framework has been proposed by An et al. [10]; however, their interpretation did not specifically address the role of OS. In contrast, more recent studies have provided additional evidence supporting the regulation of OS as an important and emerging mechanism in ADMSC‐exosome‐mediated wound healing.

### 6.1. By Inhibition of OS, ADMSC‐Exosomes Can Promote the Healing of DFU

Under diabetic conditions, persistent hyperglycemia together with local hypoxia promotes excessive mitochondrial generation of ROS [53]. Elevated ROS levels exert cytotoxic effects on keratinocytes, fibroblasts, and endothelial cells, thereby impairing neovascularization, granulation tissue formation, and extracellular matrix (ECM) deposition [54]. In addition, ROS overproduction sustains the release of proinflammatory cytokines, further aggravating endothelial dysfunction [55]. These findings highlight OS as a central driver of DFU pathogenesis. Emerging evidence indicates that ADMSC‐exosomes can enhance the proliferation, migration, and survival of skin cells under hypoxic and OS conditions [56]. They also promote collagen synthesis and angiogenesis, ultimately facilitating diabetic wound closure [57]. Mechanistically, ADMSC‐exosomes regulate the SIRT3/SOD2 signaling axis, thereby alleviating OS and inflammation, improving endothelial function, and enhancing angiogenesis [54]. Furthermore, Jiang et al. [57] demonstrated that ADMSC‐exosomes incorporated into MMP‐responsive PEG hydrogels effectively reduce intracellular ROS levels and counteract OS‐induced inhibition of cell migration, accelerating wound repair. Collectively, these findings suggest that ADMSC‐exosomes mitigate OS, restore endothelial function, and promote angiogenesis, thereby improving DFU healing outcomes.

### 6.2. ADMSC‐Exosomes Can Promote the Healing of DFU Through Anti‐Inflammation and Anti‐Infection Pathways

Chronic inflammation represents a major barrier to wound healing in DFU. Although inflammation is an essential component of host defense, its persistence in diabetic wounds leads to impaired repair and increased susceptibility to infection and amputation [58–60]. Therefore, controlling inflammation and infection is a critical therapeutic objective. Macrophages are key regulators throughout the wound healing process, participating in inflammation initiation, resolution, and tissue remodeling [58, 61]. Classically activated M1 macrophages produce proinflammatory cytokines such as IL‐1*β*, IL‐6, and TNF‐*α*, whereas alternatively activated M2 macrophages secrete anti‐inflammatory and prorepair mediators, including VEGF, IL‐10, and TNF‐*α* [58, 62]. The transition from the M1 to the M2 phenotype is essential for effective tissue regeneration [59]. ADMSC‐exosomes play an immunomodulatory role in this process. They suppress interferon alpha (IFN‐*α*) production and inhibit T‐cell activation, thereby reducing M1 macrophage accumulation [63, 64]. Additionally, they contain immunoregulatory factors such as TNF‐*α*, macrophage colony‐stimulating factor (MCSF), and RBP‐4, which can inhibit excessive inflammatory responses or correct abnormal immune reactions by influencing immune cell proliferation, differentiation, and function, contributing to the resolution of excessive inflammation [64]. The capacity of ADMSC‐exosomes to promote monocyte differentiation into M1 macrophages has been demonstrated [65]. In addition, miR‐155 carried by ADMSC‐exosomes can drive adipocyte‐derived macrophages from obese mice toward an M1 phenotype, potentially contributing to chronic inflammation through disruption of the M1‐to‐M2 balance in adipose tissue [66]. Moreover, ADMSC‐exosomes can upregulate macrophage inflammatory protein‐1*α* and monocyte chemoattractant protein‐1, thereby modulating early inflammatory responses [67]. Conversely, accumulating evidence suggests that ADMSC‐exosomes can also induce macrophage polarization toward the M2 phenotype through signaling pathways such as JAK/STAT6, enhancing macrophage viability, migration, and adhesion, and promoting neovascularization and blood perfusion in ischemic lower limbs of T2DM mice [68]. Li et al. [59] demonstrated that exosomal lncRNA H19 facilitates M2 polarization, which in turn enhances fibroblast proliferation and migration, promotes endothelial angiogenesis, and accelerates cutaneous wound healing. In vivo experiments confirm that ADMSC‐exosome treatment significantly reduces the expression of proinflammatory cytokines TNF‐*α*, IL‐6, and IL‐1*β* in diabetic wound tissues [69]. Thus, ADMSC‐exosomes improve the wound microenvironment by regulating immune responses and promoting the M1‐to‐M2 transition, thereby facilitating tissue regeneration.

### 6.3. ADMSC‐Exosomes Can Promote the Healing of DFU by Promoting Angiogenesis in Wounds

Angiogenesis is indispensable for tissue repair, as it supplies oxygen, nutrients, specific hormones, and growth factors for tissues, and enables metabolic waste removal. Thus, angiogenesis serves as a core link throughout the wound healing process. Vascular endothelial cells, fibroblasts, and vascular endothelial growth factor are regarded as crucial regulators in this biological cascade [70]. Apart from blood perfusion, newly formed blood vessels assist in the exchange of nutrients and metabolic substances to support tissue repair [71]. Mounting evidence has confirmed that ADMSC‐exosomes markedly boost angiogenic activity by stimulating the proliferation and migratory capacity of vascular endothelial cells [72]. Exosomes derived from human adipose‐derived stem cells (HADSCs) are enriched in miRNA‐125a and miRNA‐31, which can be transferred to vascular endothelial cells to promote their growth and vascular formation, as demonstrated in both in vitro and in animal experiments [73]. Furthermore, ADMSC‐exosomes facilitate endothelial tip cell migration and sprouting by suppressing antiangiogenic factors [70, 72–74]. For instance, they can inhibit the expression of the antiangiogenic gene HIF‐1*α* in vascular endothelial cells, thereby enhancing angiogenic activity [74]. Beyond vascular formation, ADMSC‐exosomes improve tissue survival under ischemic conditions by increasing capillary density and promoting skin flap viability [75]. They also enhance VEGF expression, thereby accelerating re‐epithelialization, angiogenesis, and collagen deposition in diabetic wounds [76]. Exosomes derived from adipose‐derived regenerative cells (ADRCs) have similarly been shown to improve angiogenesis and restore blood perfusion in hind‐limb ischemia [73]. ADRC‐exosomes exert anti‐inflammatory effects via the ROCK1/PTEN pathway, while promoting angiogenesis through the delivery of miR‐132 and miR‐146a mediated regulation [77]. ADMSC‐exosomes enhance proliferation, migration, and tube formation in human umbilical vein endothelial cells (HUVECs), confirming their proangiogenic potential [48, 78]. Taken together, these findings indicate that ADMSC‐exosomes promote vascular regeneration through multiple signaling pathways, thereby accelerating wound healing.

### 6.4. ADMSs‐Exosomes Can Promote the Healing of DFU by Speeding Up Proliferation and Re‐Epithelialization of Skin Cells

During the proliferative phase, fibroblasts synthesize ECM components, whereas epithelial cells proliferate and migrate to cover the wound surface. These processes are essential for effective tissue regeneration [71]. ADMSC‐exosomes are readily internalized by fibroblasts, promoting their proliferation, migration, and collagen production in a dose‐dependent manner [77]. In vivo experiments have shown that ADMSC‐exosomes accelerate cutaneous wound repair by improving fibroblast function [75]. Additionally, HADSC‐exosomes can modulate miRNA expression, upregulating 199 miRNAs and downregulating 93 miRNAs, thereby enhancing fibroblast proliferation and differentiation and promoting skin regeneration [79]. Consistent findings from other studies indicate that exosomes improve fibroblast biological behavior, leading to enhanced migration, proliferation, and ECM synthesis [80]. Yang et al. [78] showed that microRNA‐21, which is abundantly enriched in ADMSC‐exosomes. This functional miRNA can boost the migration and proliferation of HaCaT cells and facilitate wound repair by upregulating MMP‐9 expression via activation of the PI3K/AKT signaling cascade. Zhang et al. [81] further demonstrated that ADMSC‐exosomes can promote scarless skin regeneration through PI3K/AKT‐dependent sebocyte activation. Additional studies confirm that ADMSC‐exosomes enhance neovascularization, fibroblast activity, and collagen synthesis in diabetic wounds [82]. In summary, these vesicles play a crucial role in promoting cell proliferation, migration, and functional optimization during wound healing.

### 6.5. ADMSC‐Exosomes Can Promote the Healing of DFU by Regulating Collagen Remodelling to Inhibit Scar Hyperplasia

Scar hyperplasia refers to morphological and histological changes in skin and soft tissues after cutaneous wound repair. Severe tissue trauma and large‐area burns commonly trigger abnormal scar overgrowth. Such pathological changes greatly affect physical appearance and may even cause irreversible functional impairment of local tissues and organs [83]. ADMSC‐exosomes have been shown to modulate collagen remodeling, thereby reducing the risk of hypertrophic scarring. During the early stages of wound healing, they promote synthesis of Type III collagen, whereas in late stages, they suppress excessive Type I collagen deposition, contributing to improved scar quality [84]. Furthermore, ADMSC‐exosomes facilitate ECM remodeling by modulating fibroblast differentiation and gene expression profiles, thereby supporting tissue repair while limiting excessive scar formation. Wang et al. [85] reported that these exosomes suppress the transition of fibroblasts into myofibroblasts and concurrently increase the ratio of transforming growth factor‐*β*3 (TGF‐*β*3) to TGF‐*β*1 in vivo. In addition, ADMSC‐exosomes enhance the expression of matrix metalloproteinases‐3 (MMP3) in dermal fibroblasts, leading to an increased MMP3 to tissue inhibitor of metalloproteinases‐1 (TIMP1) ratio, which favors the remodelling of ECM and contributes to reduced scarring [85]. However, some studies report inconsistent findings, indicating that in diabetic mouse models, ADMSC‐exosomes may enhance Type I collagen accumulation during the later phases of wound healing, which could contribute to excessive scarring and impede optimal healing outcomes [86, 87]. These controversies are likely related to the stage‐dependent roles of both collagen dynamics and exosome activity throughout the healing process. Further studies are needed to clarify how ADMSC‐exosomes influence collagen deposition and their potential link to scar proliferation.

## 7. Summary and Prospects

In conclusion, impaired wound healing represents a major complication in diabetic patients, placing a substantial burden on healthcare systems and the economy. Physiologically, wound healing involves four stages: hemostasis, inflammation, proliferation, and remodeling [88]. These four stages are tightly regulated yet can be disrupted by multiple factors, including neuropathy, thereby compromising healing in diabetic wounds [89]. Traditional methods have limited effectiveness. As a novel type of therapy, stem cells can repair wound tissues through the exosomes they secrete. As EVs, exosomes carry biological information such as proteins and nucleic acids and participate in intercellular communication. Furthermore, they possess inherent advantages including low immunogenicity, high stability, and ease of storage. ADMSCs secrete a large number of exosomes, thereby expanding their potential applications in the therapy of DFUs and other types of wound repair. ADMSC‐exosomes can regulate the immune response and wound inflammation, and promote angiogenesis by transferring miRNA‐125a and miRNA‐31 to vascular endothelial cells. Additionally, they can stimulate the proliferation of fibroblasts and keratinocytes, and regulate collagen remodeling. These effects may regulate the secretion of related cytokines through the ERK/MAPK pathway to inhibit scar hyperplasia. With these characteristics, ADMSC‐exosomes can serve as an optimal tool to improve fat grafting outcomes, promote wound healing in DFU patients, achieve scarless cutaneous repair, and act as a carrier for combined scaffold‐based treatments. Therefore, these findings suggest that ADMSC‐exosomes have immense potential in the therapy of DFU.

However, most of the research on ADMSC‐exosomes in the therapy of DFU primarily focuses on rat/mice models and in vitro cell experiments, lacking clinical validation. Their efficacy has been suggested, but the underlying mechanisms are still largely unknown. There remains a critical issue in this field about the procancer or anticancer status of ADMSC‐exosomes. As a result, the safety and efficacy of ADMSC‐exosomes cannot be guaranteed. Moreover, the extraction, purification, and identification of exosomes from specific cell sources need to be simplified. With further studies focusing on improving the isolation and identification of ADMSC‐exosomes and elucidating their mechanisms of action, we can discover more efficient ADMSC‐exosome products and explore their broader applications in clinical practice.

**Nomenclature**
ADMSCs: adipose‐derived mesenchymal stem cells
ADMSC‐exosomes: adipose‐derived mesenchymal stem cell exosomes
DFU: diabetic foot ulcer
EV: extracellular vesicles
ECM: extracellular matrix
HADSCs: human adipose stem cells
IL‐1*β*
: interleukin 1beta
IL‐6: interleukin 6
IFN‐*α*
: interferon alpha
LEVs: large extracellular vesicles
MSCs: mesenchymal stem cells
MCSF: macrophage colony‐stimulating factor
MMP3: matrix metalloproteinases‐3
OS: oxidative stress
ROS: reactive oxygen species
SEVs: small extracellular vesicles
TNF‐*α*
: tumor necrosis factor alpha
TGF‐*β*3: transforming growth factor‐*β*3
TIMP1: tissue inhibitor of matrix metalloproteinases‐1
VEGF: vascular endothelial growth factor

## Author Contributions

Rong Zhang and Zhanshuai Song designed the study, performed data analysis, and wrote the manuscript. Yongtao Su and Kai Mou supervised the references. Juan Zhang, Wenwen Zhang, Linlin Li and Feifei Mou contributed to the in‐depth discussion of therapeutic implications and conducted technical review and language polishing of the manuscript. Rong Zhang and Zhanshuai Song have contributed equally to this work and shared first authorship.

## Funding

No funding was received for this manuscript.

## Disclosure

The authors thoroughly reviewed and approved the final manuscript, ensuring compliance with academic standards and publication requirements. All authors have agreed with its submission to BioMed Research International.

## Ethics Statement

The authors have nothing to report.

## Conflicts of Interest

The authors declare no conflicts of interest.

## Data Availability

Data are available upon reasonable request from the corresponding authors.

## References

[1] Guo Z. Y. and Li Y. , Therapeutic Strategies of Mesenchymal Stem Cell Exosomes for Diabetic Foot Ulcer, Advances in Clinical Medicine. (2023) 13, no. 5, 7934–7938, 10.12677/acm.2023.1351110.

[2] Li X. , Jing X. , Yu Z. , and Huang Y. , Diverse Antibacterial Treatments Beyond Antibiotics for Diabetic Foot Ulcer Therapy, Advanced Healthcare Materials. (2023) 12, no. 23, e2300375, 10.1002/adhm.202300375, 37141030. 37141030

[3] Sun H. , Saeedi P. , Karuranga S. , Pinkepank M. , Ogurtsova K. , Duncan B. B. , Stein C. , Basit A. , Chan J. C. N. , Mbanya J. C. , Pavkov M. E. , Ramachandaran A. , Wild S. H. , James S. , Herman W. H. , Zhang P. , Bommer C. , Kuo S. , Boyko E. J. , and Magliano D. J. , IDF Diabetes Atlas: Global, Regional and Country-Level Diabetes Prevalence Estimates for 2021 and Projections for 2045, Diabetes Research and Clinical Practice. (2022) 183, 109119, 10.1016/j.diabres.2021.109119, 34879977. 34879977 PMC11057359

[4] Ingelfinger J. R. , Armstrong D. G. , Boulton A. J. M. , and Bus S. A. , Diabetic Foot Ulcers and Their Recurrence, New England Journal of Medicine. (2017) 24, no. 376, 2367–2375, 10.1056/nejmra1615439. 28614678

[5] Boulton A. J. , Vileikyte L. , Ragnarson-Tennvall G. , and Apelqvist J. , The global Burden of Diabetic Foot Disease, Lancet. (2005) 366, no. 9498, 1719–1724, 10.1016/S0140-6736(05)67698-2. 16291066

[6] Papadopoulos K. S. , Piperi C. , and Korkolopoulou P. , Clinical Applications of Adipose-Derived Stem Cell (ADSC) Exosomes in Tissue Regeneration, International Journal of Molecular Sciences. (2024) 11, no. 25, 10.3390/ijms25115916. PMC11172884 38892103

[7] Gorecka J. , Kostiuk V. , Fereydooni A. , Gonzalez L. , Luo J. , Dash B. , Isaji T. , Ono S. , Liu S. , Lee S. R. , Xu J. , Liu J. , Taniguchi R. , Yastula B. , Hsia H. C. , Qyang Y. , and Dardik A. , The Potential and Limitations of Induced Pluripotent Stem Cells to Achieve Wound Healing, Stem Cell Research and Therapy. (2019) 10, no. 1, 1–10, 10.1186/s13287-019-1185-1, 30867069. 30867069 PMC6416973

[8] Baglio S. R. , Pegtel D. M. , and Baldini N. , Mesenchymal Stem Cell Secreted Vesicles Provide Novel Opportunities in (stem) Cell-Free Therapy, Frontiers in Physiology. (2012) 3, 3068129, 10.3389/fphys.2012.00359, 22973239. PMC3434369 22973239

[9] Shan X. , Zhang C. , Mai C. , Hu X. , Cheng N. , Chen W. , Peng D. , Wang L. , Ji Z. , and Xie Y. , The Biogenesis, Biological Functions, and Applications of Macrophage-Derived Exosomes, Frontiers in Molecular Biosciences. (2021) 8, 715461, 10.3389/fmolb.2021.715461, 34368234. 34368234 PMC8333870

[10] An Y. , Lin S. Y. , Tan X. J. , Zhu S. , Nie F. , Zhen Y. , Gu L. , Zhang C. , Wang B. , Wei W. , Li D. , and Wu J. , Exosomes From Adipose-Derived Stem Cells and Application to Skin Wound Healing, Cell Proliferation. (2021) 54, no. 3, e12993, 10.1111/cpr.12993, 33458899. 33458899 PMC7941238

[11] Kalluri R. and Lebleu V. S. , The Biology, Function, and Biomedical Applications of Exosomes, Science. (2020) 367, no. 6478, 10.1126/science.aau697. PMC7717626 32029601

[12] Alzhrani G. N. , Alanazi S. T. , Sumayyah Y. , Alsharif S. Y. , Albalawi A. M. , Alsharif A. A. , Abdel-Maksoud M. S. , and Elsherbiny N. , Exosomes: Isolation, Characterization and Biomedical Applications, Cell Biology International. (2021) 45, no. 9, 1807–1831, 10.1002/cbin.11620. 33913604

[13] Chan B. D. , Wing-Yan W. , Muk-Lan L. M. , Cho W. C. , Yee B. K. , Kwan Y. W. , and Tai W. C. , Exosomes in Inflammation and Inflammatory Disease, Proteomics. (2019) 19, no. 8, 10.1002/pmic.201800149. 30758141

[14] Min L. , Vega M. R. D. L. , Wen Q. , Bharara M. , Jiang T. , Zhang R. , Zhou S. , Wong P. K. , Wondrak G. T. , Zheng H. , and Zhang D. D. , An Essential Role of NRF2 in Diabetic Wound Healing, Diabetes. (2016) 65, no. 3, 780–793, 10.2337/db15-0564, 26718502. 26718502 PMC4764153

[15] Sun H. , Hao H. , Liu X. , Geng H. , and Liang J. , Evaluation of the Healing Potential of Short-Term Ozone Therapy for the Treatment of Diabetic Foot Ulcers, Frontiers in Endocrinology. (2023) 14, 1664–2392, 10.3389/fendo.2023.1304034, 38292773. PMC10825947 38292773

[16] Liu Z. , Xue H. , and Yang G. , A Method For Extraction of Exosomes From Breast Tumour Cells and Characterisation by Transmission Electron Microscopy, Journal of Microscopy. (2023) 292, no. 3, 117–122, 10.1111/jmi.13235, 37855326. 37855326

[17] Sokolova V. , Ludwig A. , Hornung S. , Rotan O. , Horn P. A. , Epple M. , and Giebel B. , Characterisation of Exosomes Derived From Human Cells by Nanoparticle Tracking Analysis and Scanning Electron Microscopy, Colloids and Surfaces B Biointerfaces. (2011) 87, no. 1, 146–150, 10.1016/j.colsurfb.2011.05.013, 21640565. 21640565

[18] Qi L. , Comparison of Extraction Methods of Exosomes From Patients With Ovarian Cancer, Journal of the Fourth Military Medical University. (2007) 88587192, https://api.semanticscholar.org/CorpusID.

[19] Clayton A. , Court J. , Navabi H. , Adams M. , Mason M. D. , Hobot J. A. , Newman G. R. , and Jasani B. , Analysis of Antigen Presenting Cell Derived Exosomes, Based on Immuno-Magnetic Isolation and Flow Cytometry, Journal of Immunological Methods. (2001) 247, no. 1-2, 163–174, 10.1016/S0022-1759(00)00321-5, 11150547. 11150547

[20] Bokun V. , Strang B. , Pantazi P. , Liu Y. , and Holder B. , Nano-Flow Cytometry-Guided Discrimination and Separation of Human Cytomegalovirus Virions and Extracellular Vesicles, Journal of Extracellular Vesicles. (2025) 14, no. 5, 10.1002/jev2.70060, 40314077. PMC12046292 40314077

[21] Calabro P. , Riegler L. , Maddalon V. , Fimiani F. , Limongelli G. , Martone F. , D′Alessandro R. , Ziello B. , Golino P. , and Calabro′ R. , Adipose Tissue as an Endocrine Organ: Production of Serum Amyloid a in Response to Inflammatory Cytokines by Human Adipocytes, European Heart Journal. (2013) 34, no. supplement 1, 10.1093/eurheartj/eht309.P4160.

[22] Deng Z. , Poliakov A. , Hardy R. W. , Clements R. , Liu C. , Liu Y. , Wang J. , Xiang X. , Zhang S. , Zhuang X. , Shah S. V. , Sun D. , Michalek S. , Grizzle W. E. , Garvey T. , Mobley J. , and Zhang H. G. , Adipose Tissue Exosome-Like Vesicles Mediate Activation of Macrophage-Induced Insulin Resistance, Diabetes. (2009) 58, no. 11, 2498–2505, 10.2337/db09-0216, 19675137. 19675137 PMC2768161

[23] Koeck E. S. , Iordanskaia T. , Sevilla S. , Ferrante S. C. , Hubal M. J. , Freishtat R. J. , and Nadler E. P. , Adipocyte Exosomes Induce Transforming Growth Factor Beta Pathway Dysregulation in Hepatocytes: A Novel Paradigm for Obesity-Related Liver Disease, Journal of Surgical Research. (2014) 192, no. 2, 268–275, 10.1016/j.jss.2014.06.050, 25086727. 25086727

[24] Ogawa R. , Tanaka C. , Sato M. , Nagasaki H. , Sugimura K. , Okumura K. , Nakagawa Y. , and Aoki N. , Adipocyte-Derived Microvesicles Contain RNA That Is Transported Into Macrophages and Might be Secreted Into Blood Circulation, Biochemical and Biophysical Research Communications. (2010) 398, no. 4, 723–729, 10.1016/j.bbrc.2010.07.008, 20621060. 20621060

[25] Lin R. , Wang S. , and Zhao R. C. , Exosomes From Human Adipose-Derived Mesenchymal Stem Cells Promote Migration Through Wnt Signaling Pathway in a Breast Cancer Cell Model, Molecular and Cellular Biochemistry. (2013) 383, no. 1-2, 13–20, 10.1007/s11010-013-1746-z. 23812844

[26] Toyserkani N. M. , Christensen M. L. , Sheikh S. P. , and Sørensen J. A. , Adiposederived Stem Cells: New Treatment for Wound Healing, Annals of Plastic Surgery. (2015) 75, no. 1, 117–123, 10.1097/SAP.0000000000000083. 24691309

[27] Fraser J. K. , Wulur I. , Alfonso Z. , and Hedrick M. H. , Fat Tissue: an Underappreciated Source of Stem Cells for Biotechnology, Trends in Biotechnology. (2006) 24, no. 4, 150–154, 10.1016/j.tibtech.2006.01.010. 16488036

[28] Katsuda T. , Tsuchiya R. , Kosaka N. , Yoshioka Y. , Takagaki K. , Oki K. , Takeshita F. , Sakai Y. , Kuroda M. , and Ochiya T. , Human Adipose Tissue-Derived Mesenchymal Stem Cells Secrete Functional Neprilysin-Bound Exosomes, Scientific Reports. (2013) 3, no. 1, 10.1038/srep01197, 23378928. PMC3561625 23378928

[29] Durcin M. , Fleury A. , Taillebois E. , Hilairet G. , Krupova Z. , Henry C. , Truchet S. , Trötzmüller M. , Köfeler H. , Mabilleau G. , Hue O. , Andriantsitohaina R. , Martin P. , and le Lay S. , Characterisation of Adipocyte-Derived Extracellular Vesicle Subtypes Identifies Distinct Protein and Lipid Signatures for Large and Small Extracellular Vesicles, Journal of Extracellular Vesicles. (2017) 6, no. 1, e1305677, 10.1080/20013078.2017.1305677, 28473884. PMC5405565 28473884

[30] Skotland T. , Sandvig K. , and Llorente A. , Lipids in Exosomes: Current Knowledge and the Way Forward, Progress in Lipid Research. (2017) 66, 30–41, 10.1016/j.plipres.2017.03.001. 28342835

[31] Rosenberger D. C. , Blechschmidt V. , Timmerman H. , Wolff A. , and Treede R. D. , Challenges of Neuropathic Pain: Focus on Diabetic Neuropathy, Journal of Neural Transmission. (2020) 127, no. 4, 589–624, 10.1007/s00702-020-02145-7, 32036431. 32036431 PMC7148276

[32] Wang Y. , Shi L. , Lu J. , Wang F. , Zhou Z. , Wang Y. , du X. , Qin D. , Chen F. , Shao D. , Gao Y. , Gao C. , and Sun T. , Probiotic Active Gel Promotes Diabetic Wound Healing Through Continuous Local Glucose Consumption and Antioxidant, Journal of Nanobiotechnology. (2025) 23, no. 1, 1–21, 10.1186/s12951-025-03115-5, 39885505. 39885505 PMC11780939

[33] Wang S. , Zhang Y. , Zhong Y. , Xue Y. , Liu Z. , Wang C. , Kang D. D. , Li H. , Hou X. , Tian M. , Cao D. , Wang L. , Guo K. , Deng B. , McComb D. , Merad M. , Brown B. D. , and Dong Y. , Accelerating Diabetic Wound Healing by ROS-Scavenging Lipid Nanoparticle–mRNA Formulation, Proceedings of the National Academy of Sciences. (2024) 121, no. 22, e2322935121, 10.1073/pnas.2322935121, 38771877. PMC11145207 38771877

[34] Radzieta M. , Sadeghpour-Heravi F. , Peters T. J. , Hu H. , Vickery K. , Jeffries T. , Dickson H. G. , Schwarzer S. , Jensen S. O. , and Malone M. , A Multiomics Approach to Identify Host-Microbe Alterations Associated With Infection Severity in Diabetic Foot Infections: a Pilot Study, NPJ Biofilms and Microbiomes. (2021) 7, no. 1, 10.1038/s41522-021-00202-x, 33753735. PMC7985513 33753735

[35] Versey Z. , Nizer W. S. D. C. , Russell E. , Zigic S. , DeZeeuw K. G. , Marek J. E. , Overhage J. , and Cassol E. , Biofilm-Innate Immune Interface: Contribution to Chronic Wound Formation, Frontiers in Immunology. (2021) 12, 648554, 10.3389/fimmu.2021.648554, 33897696. 33897696 PMC8062706

[36] Boniakowski A. E. , Kimball A. S. , Jacobs B. N. , Kunkel S. L. , and Gallagher K. A. , Macrophage-Mediated Inflammation in Normal and Diabetic Wound Healing, Journal of Immunology. (2017) 199, no. 1, 17–24, 10.4049/jimmunol.1700223, 28630109. 28630109

[37] Aitcheson S. , Frentiu F. , Hurn S. , Edwards K. , and Murray R. Z. , Skin Wound Healing: Normal Macrophage Function and Macrophage Dysfunction in Diabetic Wounds, Molecules. (2021) 26, no. 16, 10.3390/molecules26164917, 34443506. PMC8398285 34443506

[38] Worsley A. L. , Lui D. H. , Ntow-Boahene W. , Song W. , Good L. , and Tsui J. , The Importance of Inflammation Control for the Treatment of Chronic Diabetic Wounds, International Wound JournalVolume. (2023) 20, no. 6, 2346–2359, 10.1111/iwj.14048, 36564054. PMC10333011 36564054

[39] Mascarenhas J. V. and Jude E. B. , The Charcot Foot as a Complication of Diabetic Neuropathy, Current Diabetes Reports. (2014) 14, no. 12, 1–9, 10.1007/s11892-014-0561-6. 25354828

[40] Virtanen S. , Feskens E. , Räsänen L. , Fidanza F. , Tuomilehto J. , Giampaoli S. , Nissinen A. , and Kromhout D. , Comparison of Diets of Diabetic and Non-Diabetic Elderly Men in Finland, The Netherlands and Italy, European Journal of Clinical Nutrition. (2000) 54, no. 3, 181–186, 10.1038/sj.ejcn.1600916, 10713738. 10713738

[41] Hansen S. , Helweg S. , and Trojaborg W. , Long-Term Neurotoxicity in Patients Treated With Cisplatin, Vinblastine, and Bleomycin for Metastatic Germ Cell Cancer, Journal of Clinical Oncology. (1989) 7, no. 10, 1457–1461, 10.1200/JCO.1989.7.10.1457. 2476531

[42] Allan J. , Munro W. , and Figgins E. , Foot Deformities Within the Diabetic Foot and Their Influence On Biomechanics: A Review of the Literature, Prosthetics Orthotics International. (2016) 2, no. 40, 182–192, 10.1177/0309364615592705. 26209425

[43] Yang L. , Rong G. C. , and Wu Q. N. , Diabetic Foot Ulcer: Challenges and Future, World Journal of Diabetes. (2022) 13, no. 12, 1014–1034, 10.1177/7108526675. 36578870 PMC9791573

[44] Ivanov E. , Akhmetdhina M. , Erdiakov A. , and Gavrilova S. , Sympathetic System in Wound Healing: Multistage Control in Normal and Diabetic Skin, International Journal of Molecular Sciences. (2023) 24, no. 3, 10.3390/ijms24032045, 36768369. PMC9916402 36768369

[45] Okonkwo U. A. , Chen L. , Ma D. , Haywood V. A. , Barakat M. , Urao N. , and DiPietro L. A. , Compromised Angiogenesis and Vascular Integrity in Impaired Diabetic Wound Healing, PLoS ONE. (2020) 15, no. 4, e0231962, 10.1371/journal.pone.0231962. 32324828 PMC7179900

[46] Liu H. , Hao F. , and Chen B. , Hypoxic Adipose-Derived Stem Cell Exosomes as Carriers of miR-100-5p to Enhance Angiogenesis and Suppress Inflammation in Diabetic Foot Ulcers, Journal of Cell Communication and SignalingVolume. (2025) 19, no. 3, e70018, 10.1002/ccs3.70018. PMC12204848 40584821

[47] Yin D. and Shen G. , Exosomes From Adipose-Derived Stem Cells Regulate Macrophage Polarization and Accelerate Diabetic Wound Healing via the circ-Rps5/miR-124-3p axis, Immunity, Inflammation and Disease. (2024) 12, no. 6, e1274, 10.1002/iid3.1274. 38888351 PMC11184652

[48] Song P. , Liang Q. , Ge X. , Zhou D. , Yuan M. , Chu W. , and Xu J. , Adipose-Derived Stem Cell Exosomes Promote Scar-Free Healing of Diabetic Wounds via miR-204-5p/TGF-*β*1/Smad Pathway, Stem Cells International. (2025) 2025, 6344844, 10.1155/sci/6344844, 40018015. 40018015 PMC11865461

[49] Song Y. , You Y. , Xu X. , Lu J. , Huang X. , Zhang J. , Zhu L. , Hu J. , Wu X. , Xu X. , Tan W. , and du Y. , Adipose-Derived Mesenchymal Stem Cell-Derived Exosomes Biopotentiated Extracellular Matrix Hydrogels Accelerate Diabetic Wound Healing and Skin Regeneration, Advanced Science. (2023) 10, no. 30, e2304023, 10.1002/advs.202304023, 37712174. 37712174 PMC10602544

[50] Wang Z. , Feng C. , Liu H. , Xia Y. , Shan M. , and Hao Y. , Hypoxia-Induced Adipose Derived Stem Cells-Derived Exosomes Promote Diabetic Wound Healing Through circ-0001747/miR-199a-5p/HIF-1*α* axis, Archives of Dermatological Research. (2025) 317, no. 1, 10.1007/s00403-025-03921-9, 39987303. 39987303

[51] Bai X. , Li Y. , Wang P. , Xu Z. , Wei J. , He T. , and Han J. , Adipose Mesenchymal Stem Cell-derived Exosomes Rescue Mitochondrial Function Through SIRT1 to Improve Diabetic Wound Healing, Burns and Trauma. (2025) 13, 10.1093/burnst/tkaf017, 41089383. PMC12516946 41089383

[52] Guo E. , Wang L. , Wu J. , and Chen Q. , Exosomes From MicroRNA-125b-Modified Adipose-Derived Stem Cells Promote Wound Healing of Diabetic Foot Ulcers, Current Stem Cell Research and Therapy. (2025) 20, no. 4, 10.2174/011574888X287173240415050555. 38659271

[53] Ren S. , Chen J. , Guo J. , Liu Y. , Xiong H. , Jing B. , Yang X. , Li G. , Kang Y. , Wang C. , Xu X. , Liu Z. , Zhang M. , Xiang K. , Li C. , Li Q. , Machens H. G. , and Chen Z. , Exosomes From Adipose Stem Cells Promote Diabetic Wound Healing through the eHSP90/LRP1/AKT Axis, Cells. (2022) 11, no. 20, 10.3390/cells11203229, 36291096. PMC9600018 36291096

[54] Zhang Y. , Bai X. , Shen K. , Luo L. , Zhao M. , Xu C. , Jia Y. , Xiao D. , Li Y. , Gao X. , Tian C. , Wang Y. , and Hu D. , Exosomes Derived From Adipose Mesenchymal Stem Cells Promote Diabetic Chronic Wound Healing Through SIRT3/SOD2, Cells. (2022) 11, no. 16, 10.3390/cells11162568, 36010644. PMC9406299 36010644

[55] Tan A. , Forbes J. , and Cooper M. , AGE, RAGE, and ROS in Diabetic Nephropathy, Seminars in Nephrology. (2007) 27, no. 2, 130–143, 10.1016/j.semnephrol.2007.01.006. 17418682

[56] Kim W. , Park B. , Kim H. , Park J. S. , Kim K. J. , Choi J. S. , Chung S. J. , Kim D. D. , and Sung J. H. , Evidence Supporting Antioxidant Action of Adipose-Derived Stem Cells: Protection of Human Dermal fibroblasts From Oxidative Stress, Journal of Dermatological Science. (2008) 49, no. 2, 133–142, 10.1016/j.jdermsci.2007.08.004, 17870415. 17870415

[57] Jiang T. , Liu S. , Wu Z. , Li Q. , Ren S. , Chen J. , Xu X. , Wang C. , Lu C. , Yang X. , and Chen Z. , ADSC-exo@MMP-PEG Smart Hydrogel Promotes Diabetic Wound Healing by Optimizing Cellular Functions and Relieving Oxidative Stress, Mater Today Biology. (2022) 16, 100365, 10.1016/j.mtbio.2022.100365, 35967739. PMC9364034 35967739

[58] Raziyeva K. , Kim Y. , Zharkinbekov Z. , Kassymbek K. , Jimi S. , and Saparov A. , Immunology of Acute and Chronic Wound Healing, Biomolecules. (2021) 11, no. 5, 10.3390/biom11050700, 34066746. PMC8150999 34066746

[59] Wang Y. F. , Yang L. , Liu Y. Z. , Ma H. , Cai M. , Liang C. , Zhang L. , Su Z. , and Xu Z. , Applications and Prospects of Single-Cell RNA Sequencing and Spatial Transcriptomics in Cervical Cancer, BioMed Research International. (2025) 2025, no. 1, 1532745, 10.1155/bmri/1532745, 40612935. 40612935 PMC12226160

[60] Zhen Z. , Wei S. , Yunfei W. , Jie X. , Jienan X. , Yiting S. , Wen X. , Shuyu G. , Yue L. , Xuanyu W. , Yumei Z. , and Huafa Q. , Astragalus Polysaccharide Improves Diabetic Ulcers by Promoting M2-Polarization of Macrophages to Reduce Excessive Inflammation via the *β*-Catenin/ NF-*κ*B Axis at the Late Phase of Wound-Healing, Heliyon. (2024) 10, no. 4, e24644, 10.1016/j.heliyon.2024.e24644, 38390059. 38390059 PMC10881534

[61] Yao Y. , Xu X. H. , and Jin L. , Macrophage Polarization in Physiological and Pathological Pregnancy, Frontiers in Immunology. (2019) 10, 10.3389/fimmu.2019.00792. PMC6476302 31037072

[62] Li B. , Qian L. , Pi L. , and Meng X. , A Therapeutic Role of Exosomal lncRNA H19 From Adipose Mesenchymal Stem Cells in Cutaneous Wound Healing by Triggering Macrophage M2 polarization, Cytokine. (2023) 165, 156175, 10.1016/j.cyto.2023.156175, 36948039. 36948039

[63] Lipsky P. E. , Systemic Lupus Erythematosus: An Autoimmune Disease of B Cell Hyperactivity, Nature Immunology. (2001) 2, no. 9, 764–766, 10.1038/ni0901-764. 11526379

[64] Blazquez R. , Sanchez-Margallo F. M. , de la Rosa O. , Dalemans W. , Álvarez V. , Tarazona R. , and Casado J. G. , Immunomodulatory Potential of Human Adipose Mesenchymal Stem Cells Derived Exosomes on In Vitro Stimulated T Cells, Frontiers in Immunology. (2014) 5, no. 556, 10.3389/fimmu.2014.00556, 25414703. PMC4220146 25414703

[65] Kranendonk M. E. G. , Visseren F. L. J. , Balkom B. W. M. , Nolte-′t Hoen E. N. , van Herwaarden J. A. , de Jager W. , Schipper H. S. , Brenkman A. B. , Verhaar M. C. , Wauben M. H. , and Kalkhoven E. , Human Adipocyte Extracellular Vesicles in Reciprocal Signaling Between Adipocytes and Macrophages, Obesity. (2014) 22, no. 5, 1296–1308, 10.1002/oby.20679, 24339422. 24339422

[66] Zhang Y. , Mei H. , Chang X. , Chen F. , Zhu Y. , and Han X. , Adipocyte-Derived Microvesicles From Obese Mice Induce M1 Macrophage Phenotype Through Secreted miR-155, Journal of Molecular Cell Biology. (2016) 8, no. 6, 505–517, 10.1093/jmcb/mjw040, 27671445. 27671445

[67] Chen B. , Cai J. , Wei Y. , Jiang Z. , Desjardins H. E. , Adams A. E. , Li S. , Kao H. K. , and Guo L. , Exosomes Are Comparable to Source Adipose Stem Cells in Fat Graft Retention With Up-Regulating Early Inflammation and Angiogenesis, Plastic and Reconstructive Surgery. (2019) 144, no. 5, 816e–827e, 10.1097/PRS.0000000000006175, 31385891. 31385891

[68] Wang X. , Chen S. , Lu R. , Sun Y. , Song T. , Nie Z. , Yu C. , and Gao Y. , Adipose-Derived Stem Cell-Secreted Exosomes Enhance Angiogenesis by Promoting Macrophage M2 Polarization in Type 2 Diabetic Mice With Limb Ischemia via the JAK/STAT6 pathway, Heliyon. (2022) 8, no. 11, e11495, 10.1016/j.heliyon.2022.e11495, 36406687. 36406687 PMC9668683

[69] Zhou Y. , Zhao B. , Zhang X. L. , Lu Y. J. , Lu S. T. , Cheng J. , Fu Y. , Lin L. , Zhang N. Y. , Li P. X. , Zhang J. , and Zhang J. , Combined Topical and Systemic Administration With Human Adipose-Derived Mesenchymal Stem Cells (hADSC) and hADSC-Derived Exosomes Markedly Promoted Cutaneous Wound Healing and Regeneration, Stem Cell Research and Therapy. (2021) 12, no. 1, 10.1186/s13287-021-02287-9, 33933157. PMC8088044 33933157

[70] Kato T. , Kato K. , Shimizu Y. , Takefuji M. , and Murohara T. , Treatment With Adipose-Derived Regenerative Cells Enhances Ischemia-Induced Angiogenesis via Exosomal microRNA Delivery in Mice, Nagoya Journal of Medical Science. (2021) 83, no. 3, 465–476, 10.18999/nagjms.83.3.465, 34552283. 34552283 PMC8438007

[71] Nicholas N. , Peter J. , Alisa E. , Nissen N. N. , Polverini P. , Koch A. E. , Volin M. V. , Gamelli R. L. , and DiPietro L. A. , Vascular Endothelial Growth Factor Mediates Angiogenic Activity During the Proliferative Phase of Wound Healing, American Journal of Pathology. (1998) 152, no. 6, 1445–1452, 10.1097/00000433-199806000-00022, 9626049. 9626049 PMC1858442

[72] Martin P. , Wound Healing–Aiming for Perfect Skin Regeneration, Science. (1997) 276, no. 5309, 75–81, 10.1126/science.276.5309.75, 9082989. 9082989

[73] Zhao L. , Johnson T. , and Liu D. , Therapeutic Angiogenesis of Adipose-Derived Stem Cells for Ischemic Diseases, Stem Cell Research. (2017) 8, no. 1, 10.1186/s13287-017-0578-2. PMC5460534 28583178

[74] Liang X. , Zhang L. , Wang S. , Han Q. , and Zhao R. C. , Exosomes Secreted by Mesenchymal Stem Cells Promote Endothelial Cell Angiogenesis by Transferring miR-125a, Journal of Cell Science. (2016) 129, no. 11, 2182–2189, 10.1242/jcs.170373, 27252357. 27252357

[75] Kang T. , Jones T. M. , Naddell C. , Bacanamwo M. , Calvert J. W. , Thompson W. E. , Bond V. C. , Chen Y. E. , and Liu D. , Adipose-Derived Stem Cells Induce Angiogenesis via Microvesicle Transport of miRNA-31, Stem Cells Translational Medicine. (2016) 5, no. 4, 440–450, 10.5966/sctm.2015-0177, 26933040. 26933040 PMC4798737

[76] Pu C. M. , Liu C. , Liang J. , Yen Y. H. , Chen S. H. , Jiang-Shieh Y. F. , Chien C. L. , Chen Y. C. , and Chen Y. L. , Adipose-Derived Stem Cells Protect Skin Flaps Against Ischemia/Reperfusion Injury via IL-6 Expression, Journal of Investigative Dermatology. (2017) 137, no. 6, 1353–1362, 10.1016/j.jid.2016.12.030, 28163069. 28163069

[77] Li H. , Wang J. , Xin Z. , Zhou X. , Xiong Z. , Zhao J. , Yu R. , Huang F. , Zhang H. , and Chen L. , Exosomes Derived From Human Adipose Mensenchymal Stem Cells Accelerates Cutaneous Wound Healing via Optimizing the Characteristics of Fibroblasts, Scientific Report. (2016) 6, 10.1038/srep32993, 27615560. PMC5018733 27615560

[78] Yang C. , Bai L. , Shen X. et al., Highly-Expressed micoRNA-21 in Adipose Derived Stem Cell Exosomes Can Enhance the Migration and Proliferation of the HaCaT cells by Increasing the MMP-9 Expression Through the PI3K/AKT Pathway, Archives of Biochemistry and Biophysics. (2020) 681, 108210–108259, 10.1016/j.abb.2020.108259. 31926164

[79] Choi E. W. , Seo M. K. , Woo E. Y. , Kim S. H. , Park E. J. , and Kim S. , Exosomes From Human Adipose-Derived Stem Cells Promote Proliferation and Migration of Skin Fibroblasts, Experimental Dermatology. (2018) 27, no. 10, 1170–1172, 10.1111/exd.13451, 28940813. 28940813

[80] Zhang Y. , Zouboulis C. C. , and Xiao Z. , Exosomes From Adipose-Derived Stem Cells Activate Sebocytes Through the PI3K/AKT/SREBP-1 Pathway to Accelerate Wound Healing, Cell and Tissue Research. (2024) 396, no. 3, 329–342, 10.1007/s00441-024-03872-z. 38411945 PMC11144157

[81] Zhang J. , Yi Y. , Yang S. , Zhu Y. , and Hu X. , Effects of Adipose-Derived Stem Cell Released Exosomes on Proliferation, Migration, and Tube-Like Differentiation of Human Umbilical Vein Endothelial Cells, Chinese Journal of Reparative and Reconstructive Surgery. (2018) 32, no. 10, 1351–1357, 10.7507/1002-1892.201804016, 30600670. 30600670 PMC8414149

[82] Hade M. D. , Suire C. N. , and Suo Z. , Mesenchymal Stem Cell-Derived Exosomes: Applications in Regenerative Medicine, Cells. (2021) 8, no. 10, 10.3390/cells10081959. PMC8393426 34440728

[83] Shaito A. , Aramouni K. , Assaf R. , Parenti A. , Orekhov A. , Yazbi A. E. , Pintus G. , and Eid A. H. , Oxidative Stress-Induced Endothelial Dysfunction in Cardiovascular Diseases, Frontiers in Bioscience-Landmark. (2022) 27, no. 3, 10.31083/j.fbl2703105. 35345337

[84] Rebecca A. , Dragovic A. , Alexandra S. , Gardiner C. , Brooks A. S. , Tannetta D. S. , Ferguson D. J. , Hole P. , Carr B. , Redman C. W. , Harris A. L. , Dobson P. J. , and Harrison P. , Sizing and Phenotyping of Cellular Vesicles Using Nanoparticle Tracking Analysis, Nanomedicine: Nanotechnology Biology and Medicine. (2011) 7, no. 6, 780–788, 10.1016/j.nano.2011.04.003, 21601655. 21601655 PMC3280380

[85] Wang L. , Hu L. , Zhou X. , McGee K. C. , Harte A. L. , da Silva N. F. , Al-Daghri N. , Creely S. J. , Kusminski C. M. , Tripathi G. , Levick P. L. , Khanolkar M. , Evans M. , and Chittari M. V. , Visfatin is regulated by rosiglitazone in type 2 diabetes mellitus and influenced by NF*κ*B and JNK in human abdominal subcutaneous adipocytes, Scientific Report. (2011) 6, no. 6, e13321, 10.1371/journal.pone.0020287, 21694775. PMC3111427 21694775

[86] Wang J. , Yi Y. , Zhu Y. , Wang Z. , Wu S. , Zhang J. , Hu X. , and Nie J. , Effects of Adipose-Derived Stem Cell Released Exosomes on Wound Healing in Diabetic Mice, Chinese Journal of Reparative and Reconstructive Surgery. (2020) 34, no. 1, 124–131, 10.7507/1002-1892.201903058, 31939247. 31939247 PMC8171827

[87] Taverna S. , Pucci M. , and Alessandro R. , Extracellular Vesicles: Small Bricks for Tissue Repair/Regeneration, Annals of Translational Medicine. (2017) 5, no. 4, 10.21037/atm.2017.01.53. PMC5337202 28275628

[88] Strodtbeck F. , Physiology of Wound Healing, Newborn & Infant Nursing Reviews. (2001) 1, no. 1, 43–52.

[89] Weringer E. and Arquilla E. , Wound Healing in Normal and Diabetic Chinese Hamsters, Diabetologia. (1981) 21, no. 4, 394–401, 10.1007/BF00252688. 7286499

